# Assessment of Pelvic-Lumbar-Thigh Biomechanics to Optimize The Childbirth Position: An “*In Vivo*” Innovative Biomechanical Study

**DOI:** 10.1038/s41598-019-52338-8

**Published:** 2019-11-04

**Authors:** David Desseauve, Fabrice Pierre, Anna Fernandez, Henri Panjo, Arnaud Decatoire, Patrick Lacouture, Laetitia Fradet

**Affiliations:** 1 Department of Obstetrics and Gynecology and Reproductive Medicine, University Hospital, Poitiers University, Poitiers, France; 2 Pprime Institute - CNRS UPR 3346, Axis RoBioSS, Poitiers University, Poitiers, France; 3 Department of Obstetrics and Gynecology, Centre Hospitalier Universitaire Vaudois (CHUV), Lausanne, CH Switzerland; 4 Gender, Sexual and Reproductive Health, Centre for Research in Epidemiology and Population Health, (CESP), F-94807 Villejuif, France; 5 INED, F-75020, Paris, France

**Keywords:** Translational research, Biomedical engineering

## Abstract

The study aimed to assess the associations between the pelvis orientation, lumbar curve and thigh postures throughout pregnancy in a population of healthy women. Additionally, optimal mechanical birth conditions in terms of the pelvic inlet and lumbar curve were researched. The individuals’ posture was assessed with three-dimensional motion analysis and the lumbar curve with the Epionics SPINE system. The association between the hip joint angles (flexion and abduction), the pelvis external conjugate, and lumbar curve position was assessed with a generalized linear mixed model (GLMM) adjusted to individuals’ characteristics. Joint laxity was assessed with a modified Jobbin’s extensometer. For all of the subjects, hip flexion and hip abduction were significantly associated with the angle between the external conjugate and spine, with higher correlation in the multivariate regression model. The association between hip flexion and the lumbar curve was less significant in multivariate than univariate regression analysis. Optimal birth conditions were never reached. The findings contribute to the understanding of the association between the hip position (flexion and abduction), pelvic orientation, and lumbar curve adjusted for joint laxity in healthy pregnant women. They lay the groundwork for future research in the field of obstetrical biomechanics.

## Introduction

The rise in the caesarean section (CS) rate has been alarming, and while numerous solutions have been attempted, they have not resulted in a significant reduction in the CS rate^1^. The reasons for this continuous increase in the CS rate are multifactorial, as is extensively explained in a recent Lancet series^2^. In specific studies focusing on indications for CS, the increase in CS rates associated with the “arrest of labour” is an important contributor to this problem. An annual increase of 3.9% for this indication was highlighted by Barber *et al*.^3^. Similarly, a worrying rise in the overall CS rate at full dilatation has been noted^4,5^. Multifaceted strategies are needed to counteract this trend^6^. However, to the best of our knowledge, research on this issue has not yet been conducted in terms of obstetrical mechanical factors, and to date, this problem has not been assessed from a biomechanical point of view. This view, which considers the global posture of the parturient woman, is of utmost importance when aiming to achieve the best conditions for foetal progression. In this context, two questions need to be addressed. First, how can the methods be used to safely investigate the postures of pregnant women? Second, how can the optimal posture be defined.

Progress in movement analysis and its computationally derived methods has provided safe technological solutions that can be extrapolated to the analysis of the posture of a parturient woman^7^.

Different considerations must be taken into account regarding the definition of the optimal biomechanical conditions for giving birth. In 2015, in the western region of France, 90% of pregnant women gave birth in the supine position, and this statistic was similar to those of other industrialized countries^8^. In this context, in which the use of this position is enforced by cultural factors, anaesthetics, habits, and beliefs, it seems natural to consider optimizing the gynaecological position (on the back with the legs in stir-ups), and this optimization is a new challenge. In a recent publication, we emphasized the leading role of the pelvic orientation and lumbar curve in foetal progression^7^.

Thus, the optimized position should allow the foetal body to progress under the best conditions through the birth canal. The best conditions combine the flattening of the lumbar hinge with a pelvic inlet plane perpendicular to the axis of foetal progression.

In fact, this interrelatedness between the “channel features” (pelvis and lumbar curve) and the progression of the “passenger” was previously hypothesized by Asphasie in ancient Greece, by Faraboeuf in the nineteenth century and, more recently, by Hainault *et al*.^9–11^. These pioneering studies were complemented by radiology-based findings by Gherman *et al*. For the first time, this research assessed the effects of thigh flexion on the lumbar spine and the pelvis orientation throughout McRoberts manoeuvres^12^. From this previous work, we learned that hyperflexion of the thighs decreases lumbar lordosis as well as the cephalic rotation of 20° with respect to the pubic symphysis^12^. However, the mobilization of the thighs with the goal of achieving an optimal birthing position with an “obstetrical chute shape of the pelvic drive” remains to be addressed^13,14^.

With the addition of these factors to biomechanical considerations, the effects of joint laxity on achieving an optimal birthing position cannot be neglected. Several studies have indeed demonstrated an increase in peripheral joint laxity as pregnancy progresses^15–17^. Therefore, there is a need to determine whether joint laxity affects the relationship between thigh mobilization from one side and the lumbar spine and pelvis orientation on the other side. Since joint laxity variations due to hormonal changes have been observed during pregnancy, laxity measures need to be obtained during the different stages of pregnancy^17^.

In our study, we hypothesized that optimal foetal progression could be achieved when the pelvic inlet plane appeared to be perpendicular to the foetal body axis^7^. These optimal conditions could be obtained by mobilizing the thighs of the subject. In addition, variations in these optimal conditions during different times of pregnancy could appear mainly in regards to the variations in ligamentous laxity with respect to gestational age due to hormonal changes^17^. Thus, the main aim of this study was to assess the effects of thigh flexion and thigh abduction on the position of the pelvic inlet plane and the lumbar curve in the gynaecological position according to the individual characteristics of each pregnant woman during the 1^st^, 2^nd^ and 3^rd^ trimesters of pregnancy.

## Material and Methods

Eligible women included pregnant women who were over 18 years old, were followed during antenatal care, had a body mass index (BMI) under 40 kg.m^−2^, and had no current or previous history of inflammatory joint disease or joint hypermobility syndrome (such as Marfan’s syndrome).

Written informed consent was previously provided by each woman. The size of the sample was determined based on the parameters of typical biomechanical studies. In this case, a number of subjects above 15 appeared to have an non-significant effect on statistical power^18^. Accounting for the risk of failure in terms of the data analysis or experimentation, we defined a convenience sample of 20 pregnant women. A full protocol description is available in a recent publication of the protocol^7^. The study protocol was approved by the Ethics Committee of the Poitiers Hospital (Comité de Protection des Personnes: 2013-1203-42) and by the French National Agency of Drug Safety (Agence Nationale de Sécurité du Médicament: B131-460-22). All experiments were performed in accordance with relevant Ethics Committee and relevant French guidelines and regulations.

A traditional three-dimensional motion capture was deployed to analyse the positions of reflective markers placed on particular anatomical landmarks following a Conventional Gait Model (CGM) marker set (method described in details in our previous publication^7^). The analysis was based on an optoelectronic motion capture system using 12 infrared cameras cadenced at 100 Hz (VICON, Oxford Metrics, UK)^7^. For the pelvis, to define technical referential frames, clusters of three markers were positioned on the iliac crests. The markers located on the posterosuperior iliac spines, required to obtain pelvis and hip angles, were occulted in a supine position. Hence, their locations in these technical referential frames were established in a static standing position. Additionally, the same method was used for the markers placed on the vertebrae C7 and Th10 that were also occulted in the supine position. In their case, the technical referential frame was defined with reflective markers placed on the thorax.

The lumbar curve was assessed using the Epionics SPINE system (Epionics Medical GmbH, Potsdam, Germany)^19^ measuring the lordosis. The system consists of two flexible sensor strips using strain gauge sensors located alongside flexible circuit board strips. The positioning of the system is standardized. According to this measure, a lordosis of 0° corresponds to a perfectly flat back. The Epionics system is reliable, with the average intraclass correlation coefficient at 0.84^19^. The data acquisition (50 Hz) was transmitted in real-time via Bluetooth to a local workstation^7^.

Ligamentous laxity was estimated with a modified Jobbin’s extensometer. Its reliability was previously described as excellent with an intraclass correlation coefficient at 0.98^20^. The device measures finger joint laxity. The extension angle (°) of the metacarpophalangeal joint of the nondominant hand index finger is considered a measure of individual’s general laxity^21^. As in the case of the original Jobbin’s extensometer, the extension angle readout is performed when the torque equal to 0.26 N.m is applied to the metacarpophalangeal joint^22^.

This biomechanical study took place in an experimental setting, that is, not during labour. The environment resembled delivery room conditions as close as possible. A longitudinal assessment (sessions during the first, second, and third trimester of pregnancy) was performed for each woman included in the study. At each session, the following protocol was applied: (1) collection of data regarding the clinical characteristics of the women (height, weight, and gestational age); (2) measurement of finger laxity with the extensometer; (3) measurements of in a static supine position (5 seconds of data acquisition) with different angles of hip flexion (0°/30°/60°/90°/110°/maximum hip flexion) and abduction (30°/60°/maximum hip abduction). Each participant underwent a maximum of 18 measurement sequences in each session.

The 3D positions of the markers were tracked and collected with the optoelectronic system software package. A custom MATLAB code (MathWorks Inc., Natick, MA) was developed to merge data from the optoelectronic and the Epionics systems and to extract the required data.

Low-pass filtering using a double-pass Butterworth filter with a cutoff frequency of 10 Hz was applied to the marker positions. The CGM was used to define the hip flexion and abduction^23^. Although it has well-known limitations such as sensitivity to soft-tissue artefacts and use of Davis’s regression to determine the hip joints from external markers, the model remains widely used in clinical settings. It could be postulated that other models, such as the Calibrated Anatomical Systems Technique (CAST)^24^, could be used. However, according to Ferrari *et al*.^25^, their deployment is connected with a relatively small difference in terms of the lower body kinematics.

To describe the pelvis posture, the anatomical pelvic coordinate system was defined as proposed by Fukuchi *et al*.^26^.

**Figure 1:**
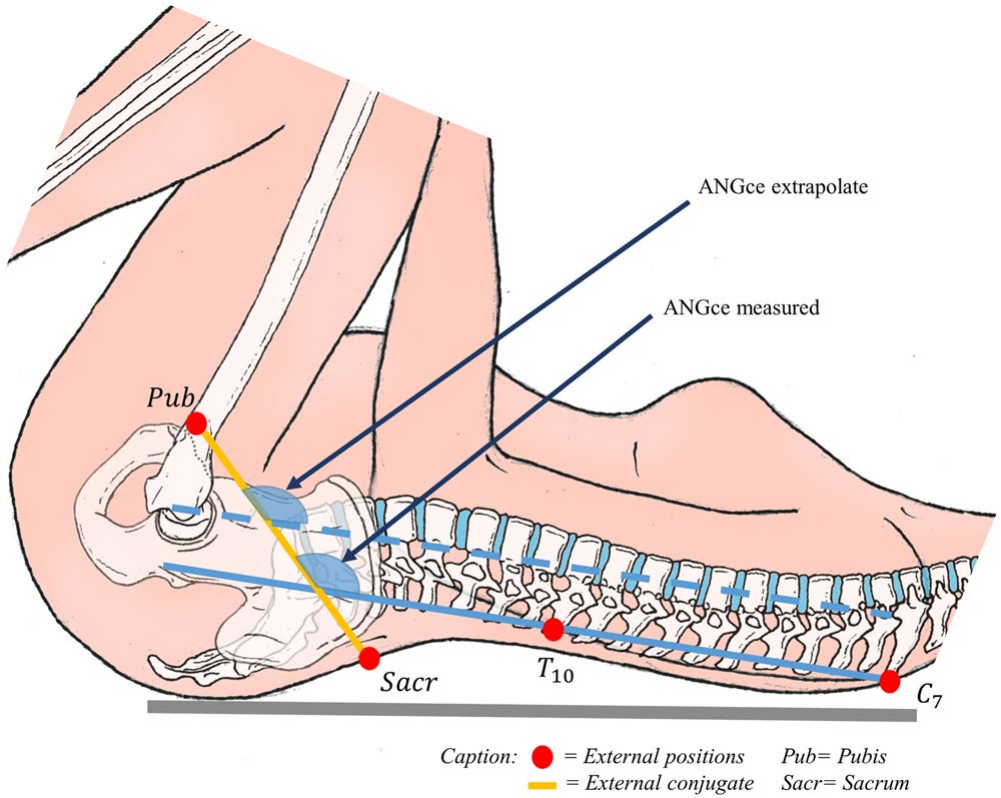
Schematic representation of the pelvic inlet plane, external conjugate, and ANGce measured in the study (acknowledgements to Marc Arcens for his illustration).

Subsequently, a plane following the external conjugate diameter was identified using the two markers located on the posterosuperior iliac spines and the marker placed on the superior edge of the pubic symphysis (Fig. 1). The flexion of the external conjugate’s plane on the spine *(ANGce)* was computed in the sagittal plane as the angle between the external conjugate and the line passing through the reconstructed markers located on the level of vertebrae C7 and Th10 (Fig. 1).

The characteristics and baseline data of the participants are described with the mean, 95% confidence interval (CI), and standard deviation for quantitative data and with the percentage for qualitative data. For BMI, we calculated the initial BMI at the first session and integrated it into other calculations in the following session. The normality of the distribution of data was estimated with the Shapiro-Wilk test.

Quarterly joint laxity comparisons were tested with the non-parametric Friedman test. If trimester was significantly associated with joint laxity, we carried out a post hoc Wilcoxon test with a Bonferroni correction.

To estimate the regression coefficient (RC) between hip flexion, hip abduction, from one side, and the *ANGce* and lumbar curve in the other side, we performed a multivariate regression. The following factors were taken into account: individual factors (age, BMI, and joint laxity), trimester of pregnancy (1^st^, 2nd or 3^rd^), and the subject effect itself. A generalized linear mixed model (GLMM) was used with a modelling covariance because this process significantly improved the likelihood ratio according to Akaike^27^. The normality of the distribution of the residues (hypothesis required for the use of the GLMM) was verified graphically by a standard normality plot (Henry’s line) in a univariate or multivariate analysis. A univariate mixed linear regression was performed to measure the associations between *ANGce*, individual parameters (maternal age, pregnancy trimesters, parity, BMI, or joint laxity), hip flexion and abduction. These parameters were all included in a multiple regression analysis. The same process was performed for the variable lumbar curve.

Since the study by Ehuis *et al*., joint laxity has been known to be correlated with age^28^. This collinearity could induce multicollinearity in the model with instability and inaccuracy in the estimation of the RC. A multicollinearity analysis with the estimation of the variance inflation factor (*vif*) was calculated for all the variables introduced in the final model. As a rule of thumb, a variable whose VIF values are greater than 10 may merit further investigation^29^.

For each woman, the conditions for optimal birth were assessed as previously proposed^13^. According to this former proposition, optimal birth is obtained when the “obstetrical chute shape of the pelvic drive” is reached, namely, when the pelvic inlet is perpendicular to the spine and when the back is flattened. In the present study, we assumed that the back was flat from −3° of lumbar lordosis or in kyphosis when values were positive. The plane of the pelvic inlet was considered perpendicular to the spine when *ANGce* reached +/−5° (in order to take into account the precision of the measures^30^). Postures (hip flexion/hip abduction and joint laxity) necessary to obtain the optimal birth conditions were assessed. To perform this analysis, we defined a binary variable that we named *“Optimac”*, this variable being set to 1 when the combination of an optimal position of the back (flat or kyphosis) and an optimal position of the pelvis (perpendicular to the lumbar spine) was obtained.

For the statistical analysis, we considered that the results were significant if p < 0.05. Statistical analysis was performed using STATA V14 software. (StataCorp, College Station, TX, USA).

## Results

This study included twenty pregnant women, 18 of whom had benefited from a quarterly evaluation. For unknown reasons, two participants were lost to follow-up between the 2^nd^ trimester and 3^rd^ trimester of pregnancy (TIP).

Based on the Friedman test and post hoc analysis, a significant difference between the TIP and joint laxity was found (Table 1).

**Table 1:** Characteristics, baseline data and joint laxity of the participants at the time of inclusion (1^st^ trimester) and subsequent evaluations (2^nd^ and 3^rd^ trimesters).

N = 18		1st T *Inclusion*	2nd T	3rdT	*P* *(Friedman)*
Maternal age	$\bar{X}$ [95% CI] - SD	33.1 [30.9–35.3] −2.8	—	—	—
Primipara	n (%)	6 (35)	—	—	—
GA (WA)	$\bar{X}$ [95% CI] - SD	13.8 [12.3–15.2] −0.7	24.0 [22.5–25.7] −0.7	34.0 [32.3–35.5] −0.7	—
BMI (kg.m^−2^)	$\bar{X}$ [95% CI] - SD	24.0 [22.0–25.0] −0.7	25.0 [24.0–28.0] −0.8	26.0 [24.0–28.0] −0.8	—
Joint Laxity	$\bar{X}$ - SD	41.3–9.0	45.3–10.8	44.04–10.0	<0.001

*BMI* = *body mass index, kg.m*^*−2*^ = *kilogram per square meter, n* = *number of subjects, GA* = *gestational age, 95% CI* = *95% confidence interval, SD* = *standard deviation, T* = *trimester, WA* = *week of amenorrhea*, $\bar{X}$  = mean.

Before adjustment, there was a significant relationship between hip flexion or hip abduction and *ANGce* for all the subjects (Table 2). Joint laxity was also correlated with *ANGce*. None of the individual factors were correlated with *ANGce*. After adjustment, hip flexion, hip abduction, joint laxity and maternal age were correlated with *ANGce*. The association was higher for hip flexion (RC = 0.23, 95% CI [0.21–0.25]) than for hip abduction (RC = 0.15, 95% CI [0.11–0.19]). Following these results, we established that 4.35° of hip flexion leads to 1° of the flexion of the pelvic inlet plane *ANGce*.

**Table 2:** Estimated correlation coefficient between *ANGce* (external conjugate) and hip position (flexion and abduction), joint laxity, and individual factors: simple and multiple regression analyses using the generalized linear mixed model (GLMM).

	*ANGce* (°): Estimation of the regression coefficient [95% CI].			
	Simple (univariate)	*P*	Multiple (multivariate)	*P*
Hips (°)				
Flexion	0.24 [0.22–0.25]	<0.001	0.23 [0.21–0.25]	<0.001
Abduction	0.26 [0.21–0.31]	<0.001	0.15 [0.11–0.19]	<0.001
Joint laxity (°)	0.35 [0.14–0.56]	<0.001	0.62 [0.45–0.79]	<0.001
Individual factors				
Age	0.39 [−0.30–1.09]	0.27	1.17 [0.15–2.20]	0.02
Pregnancy trimester	1.19 [−0.38–2.77]	0.14	−0.30 [−2.53–1.92]	0.79
BMI	0.39 [−0.39–1.18]	0.33	−0.19 [−1.06–0.67]	0.66
Nulliparity	0.72 [−5.58–7.01]	0.82	2.63 [−6.40–11.68]	0.57

*BMI* = *body mass index. kg.m*^*−2*^ = *kilogram per square meter*.

Regarding the lumbar curve analysis, hip flexion, hip abduction, and BMI were correlated with the lumbar curve (Table 3). The RC of the joint laxity was at the significance limit (*p* = 0.056). The association was higher for hip flexion than for hip abduction (RC = 0.06, 95% CI [0.05–0.07] vs RC = 0.03, 95% CI [0.01–0.05]). After adjustment for hip flexion, joint laxity, maternal age, and BMI were correlated with the lumbar curve. Individual parameters, such as maternal age (RC = 0.47, 95% CI [0.05–0.99]) and joint laxity (RC = 0.22, 95% CI [0.12–0.32]), were more correlated with lordosis than with hip flexion (RC = 0.06, 95% CI [0.05–0.07]).

**Table 3:** Estimated correlation coefficient between the lumbar curve and hip position (flexion and abduction), joint laxity, and individual factors: simple and multiple regression analyses using the generalized linear mixed model (GLMM).

	Lumbar curve (°): Estimation of the regression coefficient [95% CI].			
	Simple (univariate)	*p*	Multiple (multivariate)	*P*
Hips (°)				
Flexion	0.06 [0.05–0.07]	<0.001	0.06 [0.05–0.07]	<0.001
Abduction	0.03 [0.01–0.05]	0.016	−0.001 [−0.02–0.02]	0.92
Joint laxity (°)	0.10 [−0.01–0.20]	0.056	0.22 [0.12–0.32]	<0.001
Individual factors				
Age	0.28 [−0.07–0.63]	0.11	0.47 [0.05–0.99]	0.03
Pregnancy trimester	0.61 [−0.69–1.90]	0.36	−0.58 [−2.04–0.89]	0.44
BMI	0.69 [0.28–1.10]	0.001	0.52 [0.06–0.98]	0.03
Nulliparity	0.84 [−2.36–4.05]	0.6	0.79 [−3.37–4.95]	0.57

*BMI* = *body mass index. kg.m*^*−2*^ = *kilogram per square metre*.

The Henry curve for the multivariate models showed a normal distribution for the residues outside the extreme values.

The variance inflation factor was below 2 for each variable introduced in the model.

None of the 18 women achieved the previously defined optimal conditions. For 16 women in the second trimester and 15 women in the third trimester the optimal condition for the pelvis was never achieved while the back was flat or in kyphosis.

## Discussion

To our knowledge, this is the first study to quantify the effects of hip flexion and abduction on the pelvic orientation and lumbar curve, taking into account biomechanical parameters (joint laxity) and individual maternal characteristics.

Our results show that flexion of the thighs has a primary role and that abduction seems to play a minor role in pelvic positioning. The lumbar curve is less affected by thigh mobilization. These results can be explained by the interaction of the different musculotendinous complexes involved in hip/pelvic joint mobilization. The thighs are anatomically connected to the pelvis by a ligamentous complex. Movement of the thighs involves movement of the pelvis according to joint laxity. In contrast, the actions of thighs on the lumbar curve are less directly involved. This result might be a consequence of the divergent effects of a bi-articular muscle, the psoas muscle. Psoas muscle has an iliac and lumbar spine insertion, and each part of this muscle merges towards the femur. This muscle is then involved in hip flexion but may also increase lordosis because of its lumbar spine insertion. The action by the psoas muscle might then reduce the effects of hip flexion on the lumbar curve. The independent effects of the thighs on the pelvic orientation and lumbar curve constitute new biomechanical data that contradict current opinions on the subject, state that hip flexion mechanically causes a significant cephalad rotation of the symphysis pubis and subsequent flattening of the sacrum^12^.

Correlation analysis of these parameters shows a linear relation of 4.35 degrees hip flexion to 1-degree pelvic flexion. To our knowledge, this relation has not been previously described in the scientific literature. In clinical practice, these results emphasize the role of hip flexion in pelvic mobilization. Pelvic mobilization using hip flexion can have an impact on labour progression, as described recently by Zimerman *et al*.^31^. If hip flexion increases foetal head angle progression in the second stage of labour, our results and those of Zimerman open the door and pave the way for postural treatment when obstructed labour occurs^13^.

Our findings suggest that optimal conditions during labour, defined as the inlet plane perpendicular to the lumbar spine and the absence of a lumbar curve, were never reached by our study population. Nevertheless, we learned that 16 of 18 participants had a vaginal delivery. Thus, an optimal position is not always necessary to achieve a vaginal delivery, but finding an optimal position may help the progression of labour when labour obstruction occurs.

Individual parameters were less involved in pelvic and lumbar spine movement. Maternal age had a positive correlation with *ANGce* and the lumbar curve. With equal joint laxity, older women had higher mobilization of the pelvis and lumbar spine. This effect of maternal age on pelvic and spine mobility has to be further investigated in future studies, such as those involving hormonal effects.

The interpretation of the relationship of BMI with lordosis and not with pelvic movement is difficult to explain. One explanation could be that for women with high BMI, which is often associated with an abdominal fat belt, this abdominal supplementary weight can mechanically increase lordosis. Thus, women with a higher BMI had a higher range of lordosis reduction, and this may explain the observed relationship in our study.

Joint laxity was constantly and positively correlated with pelvic and lumbar spine outcomes. That is, greater joint laxity resulted in greater pelvic and lumbar spine mobilization. This result is inconsistent with the mechanical point of view according to which a “looser” joint results in lower intersegmental force transmission and, as such, movement imposed on a segment would have fewer effects on the adjacent segment located on the other side of the loose joint. In future research, the mechanisms involved in pelvic and lumbar mobilization according to joint laxity will need to be explored.

In this study, we investigated the biomechanics of pregnant women using a safe methodology. We demonstrated that this methodology could provide access to a holistic approach to birth positioning in further research. In the future, a better understanding of the posture conditions required to achieve an optimal birthing position could lead to customization of childbirth preparation calls, with a real effect of training on reaching the optimal birthing position if necessary.

Nevertheless, we must recognize one major limitation: this pilot study focused women who were not in labour. The next step in our research should be to confirm our results during labour.

## Conclusion

There is a linear correlation between hip position (flexion and abduction) and the pelvic orientation and lumbar curve according to joint laxity in healthy pregnant women. Independent actions of the thighs on the pelvis and lumbar curve must be assessed during labour in future research, with the objective of offering an optimal birthing position when dystocia occurs.

## Data Availability

The datasets used during the current study are available from the corresponding author on reasonable request.
